# Effects of Acute Toluene Toxicity on Different Regions of Rabbit Brain

**DOI:** 10.1155/2017/2805370

**Published:** 2017-03-26

**Authors:** Mehmet Demır, Mustafa Cicek, Nadire Eser, Atila Yoldaş, Turgay Sısman

**Affiliations:** ^1^Department of Anatomy, Faculty of Medicine, University of Kahramanmaraş, Kahramanmaraş, Turkey; ^2^Department of Medical Pharmacology, Faculty of Medicine, University of Kahramanmaraş, Kahramanmaraş, Turkey; ^3^Department of Biology, Atatürk University, Erzurum, Turkey

## Abstract

The acute phase effects of toluene on the brain have been investigated in this study using rabbit brain via histopathological, immunohistochemical, and biochemical methods. A total of 20 male rabbits were used as control and experimental groups. Moreover, nerve growth factor (NGF), tumor necrosis factor-alpha (TNF-alpha), dopamine (DA), and glial fibrillary acidic protein (GFAP) tests were performed in order to designate the severity of the biochemical damage. In the biochemical evaluation of the prefrontal cortex, hippocampus, hypothalamus, substantia nigra, and entorhinal cortex, the TNF-alpha levels in the brain were found to be significantly higher than in the control group. Levels of dopamine, secreted from the substantia nigra, nerve growth factor (NGF) developed from the hippocampal neurons, and GFAP, secreted from astrocyte cells, were detected to be significantly lower in the toluene-administration group than in the control group (*p* < 0.05). In addition, areas of focal vacuolar degeneration (abscess formation), gliosis, and perivascular demyelination, many pyknotic cells and necrosis were observed. In the toluene-administration group compared to the control group, distinct excessive expansions of the blood vessels and severe degeneration in the structure of cells and also dispersed cell borders were observed. Furthermore, abnormal malformations of the nuclei structure of the oligodendrocyte cells were seen. Bodies of the sequential neurons of the hippocampus in the toluene-administration group were distinctly structurally damaged compared to the control group. In addition, cytoplasm of the cortex cell showed serious immune reactivity in the experimental group.

## 1. Introduction

Toluene is found in crude oil and tolu trees in nature [1] and is transpired through the separation of gasoline and other fuels from crude oil and burning of coke [2, 3]. The main source of toluene exposure is gasoline, which is released to the atmosphere during production, transportation, and burning. Thus, toluene is most abundantly found in refineries, around gas stations and areas with heavy traffic. In addition, smokers are exposed to 80–100 *μ*g of toluene within a cigarette. Furthermore, top products containing toluene are paints, paint thinners, varnish, shellac, rust preventers, glues, solvent based cleaners, and some cleaning products. It is also used as a solvent in cosmetic products and commonly in benzyl chloride, benzoic acid, phenol cresol, vinyl toluene, TNT, and toluene diisocyanate production [4, 5]. Hence, many people are exposed to toluene, also via human and animal drinking waters, foods, air, and various consumer goods [1].

Toluene alters the lipid structure of the cell wall and interacts with proteins due to its lipophilic nature. In acute doses, it increases membrane fluidity by significantly elevating the Na/K-ATPase activity [6]. The correlation between the rates of the impairments it caused and dose-response relation has not yet been identified. In people and animals exposed to toluene, the first effect of toluene would be the depression of the central nervous system. In high doses, toluene has effects similar to other volatile substances such as psychomotor damage, excitation in locomotor activities and later inhibition, loss of the standing up reflex, and sedation [7–9]. Toluene induces these effects via affecting the GABAergic, glutamatergic, serotonergic, and dopaminergic pathways [10, 11]. Toluene leads to apoptotic neurodegeneration in the cerebellum and hippocampus [12]. Besides, it causes cerebellar and pyramidal dysfunctions, peripheral neuropathy, optic atrophy, neurologic hearing loss, and temporary and/or permanent damage including cognitive functions [7, 8]. Chronic toluene addiction is known to cause atrophy in the substantia alba and is associated with clinical outcomes of atrophy [13]. Moreover, it has been reported that toluene also damages the peripheral nerves [8, 14].

In humans, toluene has been found in arterial blood 10 seconds after inhalation [4]. Furthermore, when it is administered via the gastrointestinal tract, toluene reaches its maximum level in the blood in 1–3 hours. It is absorbed rather gradually by the gastrointestinal tract compared with the respiratory tract [7].

Toluene, after being absorbed, can be found, in a decreasing order, in fat tissue (white and brown adipose tissues), stomach, liver, kidneys, bone marrow, brain, and spleen [4]. Five hours after toluene is taken, it reaches the maximum level in the adipose tissue. High levels of toluene can be found in the liver and brain of people who have died of glue sniffing [15].

Prefrontal cortex is the region where feedback cycles and connections between some important sensory and motor systems that associate and integrate the top complex behavioral constituents are located. Entorhinal cortex is the part on the temporal lobe that plays a crucial role in secondary association region and memory formation. The hippocampus, on the other hand, is the region responsible for the conversion of movements to behavior, as well as memory and spatial navigation. The substantia nigra, responsible for movement and focus, which also secretes dopamine, is located in the mesencephalon [16].

Herewith, in this study, we aimed to extensively evaluate the experimental acute toxicity of toluene, a commonly encountered substance in everyday life, on the aforementioned brain regions.

## 2. Materials and Methods

This study was initiated with the permission of the Gaziosmanpaşa University Experimental Medicine Animal Experimentation Local Ethics Committee (HADYEK-51). Adult male New Zealand rabbits, weighing 4–6 kg, were used. Animals (healthy, average age 24 months) were obtained from the Gaziosmanpaşa University Experimental Medicine Research Laboratories where the study was performed. Animals were divided into Group I (toluene-administered group, *n* = 10) and Group II (control group, *n* = 10). The subjects of Group I were given a single dose of 876 mg/kg intraperitoneal (IP) toluene (99.9% solution, IRMA, Sigma-Aldrich) injection. Blood samples were collected from the animals 5 hours after the injection. Then, they were centrifuged and stored in a freezer at −80°C after serum and plasma samples were separated. The animals were euthanized via anesthetic exsanguination using the combination of 10 mg/kg xylazine (Ketalar™, Eczacıbaşı, Istanbul, Turkey) and 100 mg/kg ketamine HCl (Ketalar, Eczacıbaşı, Istanbul, Turkey). The brain tissues were removed from the cavum cranii. Random samples of the brain tissue from each group were fixed in 10% formalin for 3–6 h, dehydrated, embedded in paraffin, sectioned at 5 *μ*m, and subsequently stained with hematoxylin/eosin and immunohistochemical methods. The prepared slides were stained as described [17]. The stained slides were examined under a Research Microscope Olympus BX-50. From the obtained serum and plasma samples from the animals, the nerve growth factor (NGF), tumor necrosis factor-alpha (TNF-alpha), dopamine, and glial fibrillary acidic protein (GFAP) levels were determined using ELISA method [6].

Numerical data is shown as arithmetic mean ± standard deviation (SD). Statistical evaluation was performed via SPSS 15.0 software package program (SPSS Inc., Chicago, IL, USA). The difference between the groups was assessed with Kruskal-Wallis test and its significance with Student's *t*-test. Values of *p* < 0.05 were considered significant.

## 3. Results

### 3.1. Biochemical Findings

In this study, TNF-alpha levels were found to significantly increase in the toluene-administered group compared with the control group by using ELISA (Table 1) (*p* < 0.001).

It was seen that the dopamine (DA) serum levels were determined to be significantly reduced in the toluene-administered group compared with the control group (*p* < 0.001). Furthermore, the nerve growth factor (NGF) secreted by the hippocampal neurons was found in significantly lower levels in the toluene-administered group than in the control group (*p* < 0.001). However, the GFAP secreted from the astrocyte cells of the brain were determined to be significantly lower in the toluene-administered group compared to the control group (*p* < 0.001) (Table 1).

#### 3.1.1. Histopathological Findings

**Figure 1:**
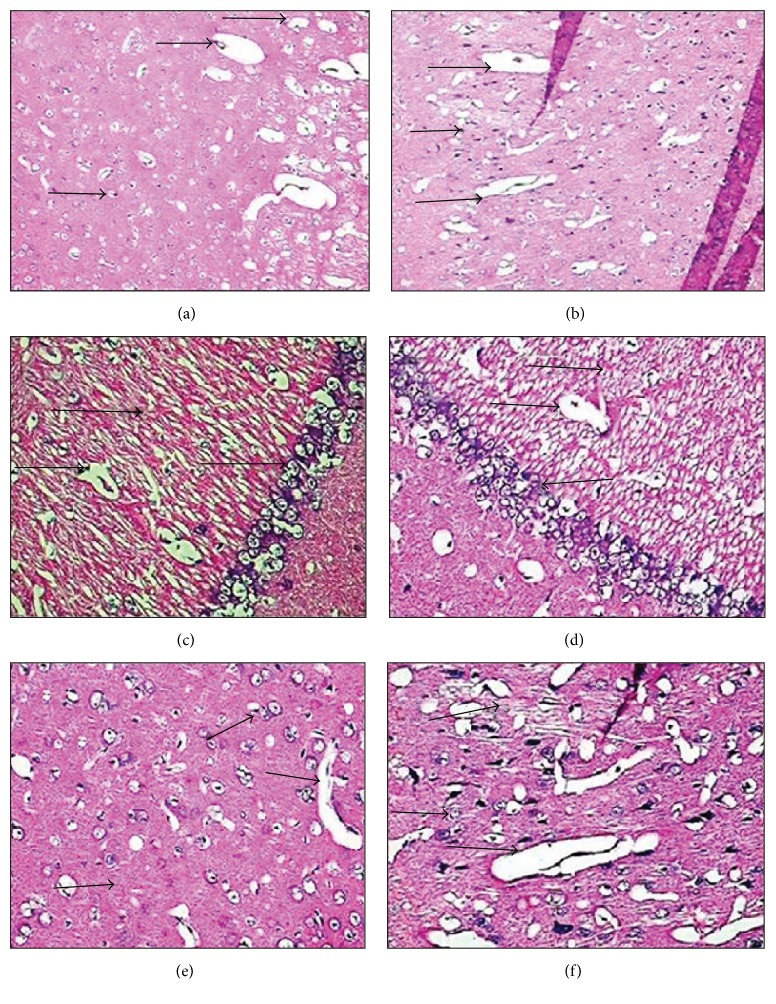
H&E stained. (a) Control group, entorhinal cortex, H&E (×20). (b) Toluene-administered group, entorhinal cortex, H&E (×20). (c) Control group, hippocampal area, H&E (×40). (d) Toluene-receiving group, hippocampal area, H&E (×40). (e) Control group, prefrontal cortex, H&E (×40). (f) Toluene-administered group, prefrontal cortex, H&E (×40).

As hematoxylin-eosin stained slides were investigated, brain tissues of the control group were observed in normal structure. Areas of focal vacuolar degeneration, gliosis, perivascular demyelination, and many pyknotic cells and necrosis were detected in the brain cortexes of the toluene-administered rabbits. In the group exposed to toluene, compared to the control group, remarkable excessive expansion of the blood vessels, severe degeneration of the compensation in the cells, and almost dispersed cell borders were observed. In the toluene-receiving group, abnormal malformations of the nuclei structure of the oligodendrocyte cells were seen. Bodies of the sequential neurons of the hippocampus in the toluene-administered group were distinctly structurally damaged compared to the control group (Figure 1).

#### 3.1.2. Immunohistochemical Findings

In this study, in the immunohistochemical staining performed on the brain tissue section of both groups in order to determine the apoptosis status using by the Bax C3 immunoreactivity was evaluated. A semiquantitative evaluation of the density of the detected reaction was performed from the staining that made Bax and C3 proteins apparent.

Cytoplasm of the cells of brain cortex, hippocampal area, entorhinal cortex and substantia nigra areas, and Bax and C3 immunoreactivity was determined negatively in the brain tissue section of the control group (0).

**Figure 2:**
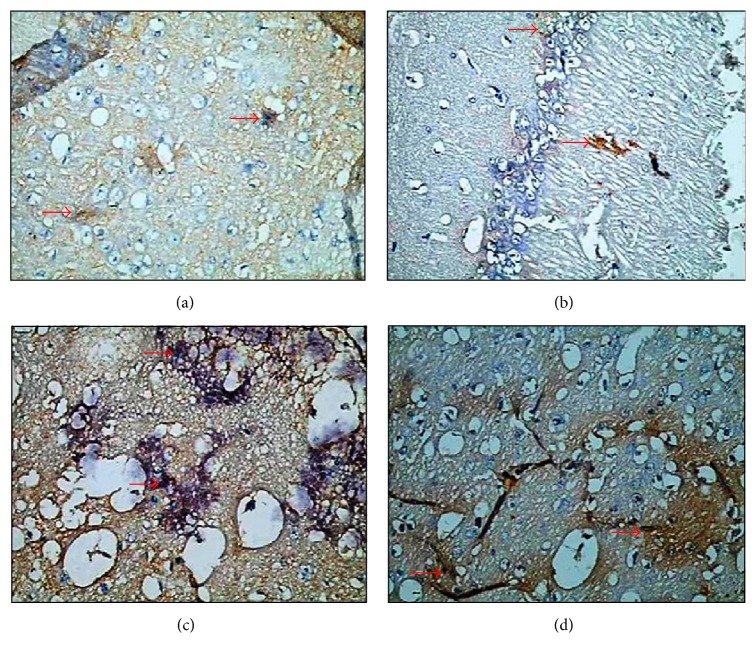
Immunohistochemical staining. (a) Control group, Bax immunoreactivity (×40). (b) Control group, C3 immunoreactivity (×40). (c) Toluene-administered group, Bax immunoreactivity (×40). (d) Toluene-administered group, C3 immunoreactivity (×40).

The immunoreactivity of Bax (3+) and C3 showed a dramatic increase in the brain cortex cell cytoplasm of the toluene-administered rabbits (Figure 2).

## 4. Discussion and Conclusion

In various in vivo studies, it is reported that, during toluene related toxic damage in the central nervous system, the cell response is seen as the first apparent response in the astrocytes. It was found that toluene shows its neurotoxic effect by increasing cholinergic activity binding to GABA receptors and shows a noncompetitive antagonistic effect against the NMDA receptors [18, 19]. In a patient who has been taking toluene for 10 years, it was seen that chronic toluene usage might have led to demyelization and axonal degeneration [19]. In rats, toluene led to oxidative stress and by increasing reactive oxygen output caused neuronal damage and gliosis. In rat studies, GFAP have also been used for determination of gliosis caused by toluene. Gliosis was found to increase dramatically in the hippocampus, cortex, and cerebellum [20, 21]. In this study, the TNF-alpha level in the toluene-administered group was significantly higher than in the control group in the biochemical evaluation of the prefrontal cortex, hippocampus, hypothalamus, substantia nigra, and entorhinal cortex. Dopamine (DA) serum levels secreted from the substantia nigra were found to significantly decrease in the toluene-administered group compared with the control group. Levels of the nerve growth factor (NGF) developed from hippocampal neurons were found to significantly reduce in the toluene-administered group compared with the control group. Levels of GFAP secreted from astrocyte cells of the brain were also determined to go down and this difference was found significant reduction as compared with the control group (*p* < 0.05).

As we evaluated the hematoxylin-eosin slides, brain tissues in the control group showed a normal structure. However, in the toluene-administered animals, areas of focal vacuolar degeneration (abscess formation), gliosis, perivascular demyelination, and many pyknotic cells and necrosis were observed in the brain cortex. Moreover, in the toluene-administered group, distinct excessive expansions of the blood vessels and severe degeneration of the compensation in the cells and dispersed cell borders were detected as compared with the control group. Nuclei of the oligodendrocyte cells in the toluene-administered group showed abnormal malformations. As compared with the control group, bodies of the sequential neurons of the hippocampus in the toluene-administered group were distinctly structurally damaged.

Apoptosis is programmed cell death characterized by the retreat of cytoplasm, membrane alterations, and DNA fracture without harming nearby cells [22]. Apoptotic pathways are generally considered as intrinsic (mitochondria mediated) and extrinsic (receptor mediated). Mitochondria mediated apoptosis is triggered by the secretion of apoptogenic factors such as cytochrome C, apoptosis inducing factor, and Smac/DIABLO from the mitochondria intermembrane space to the cytosol. Once the transport of cytochrome C to the cytosol occurs, the apoptosome complex comprised of cytochrome C/Apaf-1/ATP/procaspase-9 activates caspase-9 and then caspase-3 [23–25]. The elevation in the apoptosis mediator proapoptotic proteins (Bax, Bad, and B1d) enhances apoptosis, while the increase of antiapoptotic proteins (Bcl-2) reduces apoptosis by suppression.

Some researchers reported that toluene causes tissue damage by increasing the oxygen radicals [26, 27]. According to another hypothesis, toluene increases membrane fluidity by altering the lipid structure of the cell membrane and then affecting the Na/K-ATPase activity [6]. Toluene is also suggested to affect the GABAergic, glutamatergic, serotonergic, and dopaminergic pathways in high doses [10, 11]. Nevertheless, the latest studies indicated that toluene increases the apoptotic activity in the tissue [17, 28]. In this study, Bax and C3 immunoreactivity was reviewed in order to the find out the presence of apoptosis. The cytoplasm of the cells of brain cortex, hippocampal area, entorhinal cortex and substantia nigra areas, Bax and C3 immunoreactivity were determined negative (0). Conversely, in toluene-administered rabbits, the cell cytoplasm of brain cortex showed increased serious immunoreactivity against Bax (+3).

El-Nabi Kamel and Shehata reported that the brain tissue is the most affected part by toluene. They presented a significant increase in the caspase-3 activity, especially in the brain cortex [29]. It has been shown that toluene increases the caspase-3 activity by damaging the frontal cortex and brain stem [30]. Tas et al. [17] demonstrated that chronic exposure to toluene increases in Bax immunoreactivity. It has been suggested that it leads to a significant increase in the TUNEL positive cell count in the lung, brain, and testicular tissues of rats exposed to toluene [17, 31–33]. In our study, negative (0) caspase-3 immunoreactivity was observed in the cell cytoplasm in the areas of brain cortex, hippocampal area, entorhinal cortex, and substantia nigra in the control group. However, in the toluene-administered rabbits, brain cortex cell cytoplasm caspase-3 immune reactivity (+++) was significantly amplified as indicated by authors [17, 31–33].

As a result of this study, the main damage of toluene, which is astrocyte activation and gliosis, has been distinctly observed by making benefit of biochemical and histopathological methods. High levels of the proapoptotic proteins, Bax and caspase-3, in the brain cortex, hippocampal area, entorhinal cortex, and substantia nigra tissues of the toluene-administered rabbits showed that toluene may trigger apoptosis even in about 3 hours.

## Figures and Tables

**Table 1:** TNF-alpha, dopamine, NGF, and GFAP serum levels.

	Groups		*t*	*p*
	Control	Toluene receiving		
	Mean ± SD	Mean ± SD		
TNF-alpha	323,02 ± 3.72856	582.24 ± 12.41	63.298	0.001^*∗*^
Dopamine	6.94 ± 1.10099	4.42 ± 0.68	6.134	0.001^*∗*^
NGF	47.12 ± 3.07560	23.09 ± 4.59	13.745	0.001^*∗*^
GFAP	12.22 ± 1.26635	8.06 ± 1.89	5.766	0.001^*∗*^

^*∗*^Significant differences between the control and the toluene receiving groups. The level of significance was set as *α* = 0,05; independent sample t-test.

## References

[1] Agency for Toxic Substances and Disease Registry (ATSDR) (2006). *Case Studies in Environmental Medicine*.

[2] Agency for Toxic Substances and Disease Registry (ATSDR) (2007). *CSEM Toluene Toxicity: Environmental Medicine Case Study*.

[3] Agency for Toxic Substances and Disease Registry (ATSDR) (2000). *Toxicological Profile for Toluene*.

[4] Health Protection Agency (HPA) Toluene Toxicological Overview. http://www.hpa.org.uk/web/HPAwebFile/HPAweb_C/1194947395545.

[5] Baydas G., Gursu M. F., Yilmaz S. (2002). Daily rhythm of glutathione peroxidase activity, lipid peroxidation and glutathione levels in tissues of pinealectomized rats. *Neuroscience Letters*.

[6] Calderón-Guzmán D., Espitia-Vázquez I., López-Domínguez A. (2005). Effect of toluene and nutritional status on serotonin, lipid peroxidation levels and NA+/K+-ATPase in adult rat brain. *Neurochemical Research*.

[7] Uzun N., Karaali S. F., Kiziltan M. E. (2001). Peripheral nerve system damage in chronic toluene and n-hexane intoxication: electrophysiologic investigation. *Cerrahpaşa Journal of Medicine*.

[8] Coşkun Ö., Yüncü M., Kanter M., Büyükbaş S. (2006). Ebselen protects against oxidative and morphological effects of high concentration chronic toluene exposure on rat sciatic nerves. *European Journal of General Medicine*.

[9] Baydas G., Reiter R. J., Nedzvetskii V. S. (2003). Melatonin protects the central nervous system of rats against toluene-containing thinner intoxication by reducing reactive gliosis. *Toxicology Letters*.

[10] Meydan S., Altas M., Nacar A. (2012). The protective effects of omega-3 fatty acid against toluene-induced neurotoxicity in prefrontal cortex of rats. *Human and Experimental Toxicology*.

[11] Bowen S. E., Batis J. C., Mohammadi M. H., Hannigan J. H. (2005). Abuse pattern of gestational toluene exposure and early postnatal development in rats. *Neurotoxicology and Teratology*.

[12] Ladefoged O., Hougaard K. S., Hass U. (2004). Effects of combined prenatal stress and toluene exposure on apoptotic neurodegeneration in cerebellum and hippocampus of rats. *Basic and Clinical Pharmacology and Toxicology*.

[13] Aydin K., Sencer S., Demir T., Ogelb K., Tunaci A., Minareci O. (2002). Cranial MR findings in chronic toluene abuse by inhalation. *American Journal of Neuroradiology*.

[14] Coskun O., Oter S., Korkmaz A., Armutcu F., Kanter M. (2005). The oxidative and morphological effects of high concentration chronic toluene exposure on rat sciatic nerves. *Neurochemical Research*.

[15] U.S. Environmental Protection Agency (EPA) (2005). *Toxicological Review of Toluene*.

[16] Snell R. S. (2004). *Clinical Anatomy for Medical Students. Yıldırım M. (Çev. Ed.) s.229*.

[17] Tas U., Ogeturk M., Meydan S. (2011). Hepatotoxic activity of toluene inhalation and protective role of melatonin. *Toxicology and Industrial Health*.

[18] Eisenberg D. P. (2003). Neurotoxicity and mechanism of Toluene abuse. *Quarterly Journal of Biology and Medicine*.

[19] Sakai T., Honda S., Kuzuhara S. (2000). Encephalomyelopathy demonstrated on MRI in a case of chronic toluene intoxication. *Clinical Neurology*.

[20] Gotohda T., Tokunaga I., Kubo S.-I., Morita K., Kitamura O., Eguchi A. (2000). Effect of toluene inhalation on astrocytes and neurotrophic factor in rat brain. *Forensic Science International*.

[21] Georganopoulou D. G., Chang L., Nam J.-M. (2005). Nanoparticle-based detection in cerebral spinal fluid of a soluble pathogenic biomarker for Alzheimer's disease. *Proceedings of the National Academy of Sciences of the United States of America*.

[22] Saraste A., Pulkki K. (2000). Morphologic and biochemical hallmarks of apoptosis. *Cardiovascular Research*.

[23] Bender L. M., Morgan M. J., Thomas L. R., Liu Z., Thorburn A. (2005). The adaptor protein TRADD activates distinct mechanisms of apoptosis from the nucleus and the cytoplasm. *Cell Death and Differentiation*.

[24] Fulda S., Debatin K.-M. (2006). Resveratrol modulation of signal transduction in apoptosis and cell survival: a mini-review. *Cancer Detection and Prevention*.

[25] Friedlander R. M. (2003). Apoptosis and caspases in neurodegenerative diseases. *New England Journal of Medicine*.

[26] Karabulut I., Balkanci Z. D., Pehlivanoglu B., Erdem A., Fadillioglu E. (2009). Effect of toluene on erythrocyte membrane stability under in vivo and in vitro conditions with assessment of oxidant/antioxidant status. *Toxicology and Industrial Health*.

[27] Lee J. I., Lee K. S., Paik Y.-H. (2003). Apoptosis of hepatic stellate cells in carbon tetrachloride induced acute liver injury of the rat: analysis of isolated hepatic stellate cells. *Journal of Hepatology*.

[28] Gotohda T., Nishimura A., Morita K. (2009). Immunohistochemical studies on early stage of hepatic damage induced by subacute inhalation of toluene vapor in rats. *Journal of Applied Toxicology*.

[29] El-Nabi Kamel M. A., Shehata M. (2008). Effect of toluene exposure on the antioxidant status and apoptotic pathway in organs of the rat. *British Journal of Biomedical Science*.

[30] Kanter M. (2008). Protective effects of *Nigella sativa* on the neuronal injury in frontal cortex and brain stem after chronic toluene exposure. *Neurochemical Research*.

[31] Kanter M. (2011). Thymoquinone reestablishes spermatogenesis after testicular injury caused by chronic toluene exposure in rats. *Toxicology and Industrial Health*.

[32] Kanter M. (2011). Thymoquinone attenuates lung injury induced by chronic toluene exposure in rats. *Toxicology and Industrial Health*.

[33] Kanter M. (2011). Protective effects of thymoquinone on the neuronal injury in frontal cortex after chronic toluene exposure. *Journal of Molecular Histology*.

